# DISTMIX: direct imputation of summary statistics for unmeasured SNPs from mixed ethnicity cohorts

**DOI:** 10.1093/bioinformatics/btv348

**Published:** 2015-06-09

**Authors:** Donghyung Lee, T. Bernard Bigdeli, Vernell S. Williamson, Vladimir I. Vladimirov, Brien P. Riley, Ayman H. Fanous, Silviu-Alin Bacanu

**Affiliations:** ^1^Department of Psychiatry, Virginia Institute for Psychiatric and Behavioral Genetics, Virginia Commonwealth University, Richmond, VA 23298, USA,; ^2^Center for Biomarker Research & Personalized Medicine, Virginia Commonwealth University, Richmond, VA 23298, USA and; ^3^Lieber Institute for Brain Development, Johns Hopkins University, Baltimore, MD 21205, USA

## Abstract

**Motivation:** To increase the signal resolution for large-scale meta-analyses of genome-wide association studies, genotypes at unmeasured single nucleotide polymorphisms (SNPs) are commonly imputed using large multi-ethnic reference panels. However, the ever increasing size and ethnic diversity of both reference panels and cohorts makes genotype imputation computationally challenging for moderately sized computer clusters. Moreover, genotype imputation requires subject-level genetic data, which unlike summary statistics provided by virtually all studies, is not publicly available. While there are much less demanding methods which avoid the genotype imputation step by directly imputing SNP statistics, e.g. *D*irectly *I*mputing summary *ST*atistics (DIST) proposed by our group, their implicit assumptions make them applicable only to ethnically homogeneous cohorts.

**Results:** To decrease computational and access requirements for the analysis of cosmopolitan cohorts, we propose DISTMIX, which extends DIST capabilities to the analysis of mixed ethnicity cohorts. The method uses a relevant reference panel to directly impute unmeasured SNP statistics based only on statistics at measured SNPs and estimated/user-specified ethnic proportions. Simulations show that the proposed method adequately controls the Type I error rates. The 1000 Genomes panel imputation of summary statistics from the ethnically diverse Psychiatric Genetic Consortium Schizophrenia Phase 2 suggests that, when compared to genotype imputation methods, DISTMIX offers comparable imputation accuracy for only a fraction of computational resources.

**Availability and implementation:** DISTMIX software, its reference population data, and usage examples are publicly available at http://code.google.com/p/distmix.

**Contact:** dlee4@vcu.edu

**Supplementary information:** Supplementary Data are available at *Bioinformatics* online.

## 1 Introduction

Genotype imputation methods (Browning and Browning, 2007; Howie *et al.*, 2009; Li *et al.*, 2010; Nicolae, 2006; Servin and Stephens, 2007) are commonly used to increase the genomic resolution for large-scale multi-ethnic meta-analyses (Ripke *et al.*, 2014; Sklar *et al.*, 2011; Sullivan *et al.*, 2013) by predicting genotypes at unmeasured markers based on cosmopolitan reference panels (e.g. 1000 Genomes (1KG) (Altshuler *et al.*, 2010)). While there have been improvements (Delaneau *et al.*, 2014; Howie *et al.*, 2011; Liu *et al.*, 2013; O'Connell *et al.*, 2014; Pasaniuc *et al.*, 2010) in delivering more accurate and reliable estimation in cosmopolitan cohorts, these approaches still have two major limitations. The first is the computational burden—they are computationally very demanding due to i) their requirement of estimating haplotypes (pre-phasing) of all subjects in the study and ii) the use of large and diverse panels. For large consortium meta-analyses [e.g. Psychiatric Genetic Consortium Schizophrenia Phase 2 (PGC SCZ2) (Ripke *et al.*, 2014) and Genetic Investigation of ANthropometric Traits (Allen *et al.*, 2010)], (multiple iterations of) genotype imputation can be extremely burdensome computationally. The second limitation is the requirement for individual-level genotype data. Unlike freely available summary statistics, there is a limited (or, at least, not timely) access to genotypic data that is required by genotype imputation methods. This, in turn, might slow the process of scientific discovery.

To overcome the above mentioned limitations associated with genotype imputation methods, recently two summary statistics based imputation methods, DIST (Lee *et al.*, 2013) (developed by our group) and ImpG (Pasaniuc *et al.*, 2014), have been proposed. Both methods can directly impute summary statistics (two-tailed *Z*-scores) for unmeasured SNPs from genome-wide association studies (GWASs) consisting of both family and independent cohorts. The methods were shown to (i) substantially reduce the computational burden and (ii) be practically as accurate as commonly used genotype imputation methods. These methods were successfully applied in gene-level joint testing of functional variants using only summary data (Lee *et al.*, 2015) and functional enrichment analyses (Pickrell, 2014). However, in their present form, direct imputation methods are only amenable for imputation in ethnically homogeneous cohorts.

To extend methods like DIST to cosmopolitan cohorts additional study information might be needed, e.g. as described in Methods, cohort allele frequencies (AFs). However, it is possible to determine whether an individual subject is a study member by using only the subject's genotypes and in-cohort study AFs (Homer *et al.*, 2008). Based on this finding, to protect privacy, funding agencies, besides genotypic data, also restricted public access to GWAS AFs. However, subsequently, it was shown (Visscher and Hill, 2009) that the power of the subject identification is roughly proportional to the ratio of the number of independent loci to the number of subjects in a study cohort. Their simulations showed that even for the smaller GWAS/meta-analyses with 10 000 subjects, the detection power falls below 0.5. Even more, all these calculations assume homogeneous populations. For mixed ethnicity cohorts the population stratification encompassed in AFs estimates further confounds the subject identification. Thus, for a large cosmopolitan meta-analysis, e.g. PGC SCZ2 (>80 000 subjects), the identification power is practically negligible. Thus, for the ever increasing sizes and ethnic diversities of genetic meta-analyses, the restrictions on AF can be lifted without harming subject privacy.

In this article, we extend DIST imputation method/software to *D*irectly *I*mputing summary *ST*atistics for unmeasured SNPs from *MIX*ed ethnicity cohorts (DISTMIX). DISTMIX inherits the main advantages of DIST, i.e. speed, not requiring genetic data access and applicability to pedigree data, while gaining the capability to accurately impute association summary statistics from multi-ethnic studies.

This is achieved by (i) predicting a study’s proportions (weights) of ethnicities from a multi-ethnic reference panel based only on (common) Single Nucleotide Polymorphisms (SNPs) AFs from the studied cohort or taking, as the weights, user-specified ethnic proportions drawn from one's prior information on ethnic composition of the study samples, (ii) computing ethnicity-weighted correlation matrix based on the estimated/user-specified weights and genotypes of ethnicities from the reference panel and then (iii) using the weighted correlation matrix in a DIST procedure.

## 2 Methods

### 2.1 DISTMIX imputation

Assume that the reference panel consists of *N* ethnic groups and that the vector *G* for individual genotypes in the study cohort is a mixture of random genotypes from (not admixed from) the *N* ethnic groups with weight vector $W={\left[w_{i}\right]}_{N\times 1}$. The genotype vector *G *then follows a mixture distribution $p(G)=\Sigma_{i=1}^{N}w_{i}p(G|i)$, where p(G|i) is the genotype distribution of the *i*th ethnic group, assumed to have genotype mean $\mu_{i}$ and variance-covariance matrix $C_{i}$. By the law of total expectation and total variance/covariance, the unconditional expectation and variance-covariance matrix of *G* can be derived as

(1) $$\mu =E(G)=\Sigma_{i=1}^{N}w_{i}\mu_{i}$$

and

(2) $$C=Cov(G)=\Sigma_{i=1}^{N}w_{i}C_{i}+\Sigma_{i=1}^{N}w_{i}(\mu_{i}-\mu ){\left(\mu_{i}-\mu \right)}^{T}$$

respectively.

Let *S* be the vector of the ‘estimated study population’ SNP reference allele frequencies (RAFs), e.g. the weighted mean of case and control frequencies using the studied condition prevalence and its complement as weights, respectively. Let $P={\left[P_{i}\right]}_{1\times N}$ be the RAF matrix of the reference population ethnicities for the measured SNPs, where $P_{i}$ is the RAF vector of the *i*th ethnicity of the reference panel. By dividing Equation (1) by a factor of 2, the study cohort RAF vector can be expressed as a weighted sum of RAF vectors of reference population ethnicities with $W={\left[w_{i}\right]}_{N\times 1}$: $S=\Sigma_{i=1}^{N}w_{i}P_{i}$. After straightforward algebraic manipulations:

$$Cov(P,S)=Cov(P,\Sigma_{i=1}^{N}w_{i}P_{i})=Cov(P,PW)=Cov(P)W.$$

Linear/quadratic programming methods can be employed to estimate W subject to constraints $\Sigma_{i=1}^{N}w_{i}=1$ and $0\leq w_{i}\leq 1$. However, due to the large number of SNPs, even simply solving the linear system without constraints ($\hat{W}=Cov{\left(P\right)}^{-1}Cov(P,S)$) and substituting zero for the (very) few small negative proportions, can yield very accurate results which practically meet the weight constraints. Not using the linear/quadratic programming might even yield advantages when the reference panel does not provide good proxies for all ethnic groups in the cohort. Under such a scenario, after setting to zero negative weights, the sum of the resulting weights is likely to exceed one. In turn, this acts as an extra ridge penalty for the correlation matrix (see 3 paragraphs below), which help control DISTMIX false positive rates.

Due to the strong LD among SNPs, the calculation of the correlation using all SNPs in a genome might lead to a poor estimation. To avoid this, we sequentially split GWAS SNPs into 1000 non-overlapping SNP sets, e.g. first set consists of the 1st, 1001st, 2001st, etc. map ordered SNPs in the study. The large distances between SNPs in the same set, makes them quasi-independent which, thus, improves the accuracy of the estimated correlation. $\hat{W}$ is subsequently estimated as the average of the weights obtained from the 1000 SNP sets.

Typically, the study AF information is not publicly accessible. Thus, to make DISTMIX applicable even to summary data sets lacking AF information, we added an option for users to pre-specify the weights based on their prior knowledge on ethnic composition of the study cohort of interest. This option should be most useful when (i) fairly accurate proportion information about ethnicities involved in the cohort is available and (ii) all ethnicities in the cohort have reasonably close proxies in the reference panels.

Based on estimated/pre-specified weights $\hat{W}={\left[{\hat{w}}_{i}\right]}_{N\times 1}$, we estimate the cohort genotype correlation matrix Σ in a three step process. First, by using Equation (2), estimate the cohort genotype covariance matrix C in the sliding window as

$$\hat{C}=\Sigma_{i=1}^{N}{\hat{w}}_{i}{\hat{C}}_{i}+\Sigma_{i=1}^{N}{\hat{w}}_{i}({\hat{\mu }}_{i}-\hat{\mu }){\left({\hat{\mu }}_{i}-\hat{\mu }\right)}^{T}$$

where ${\hat{\mu }}_{i}$ and ${\hat{C}}_{i}$ are the estimated genotype mean (twice the RAF) vector and variance-covariance matrix for the *i*th ethnic group respectively and $\hat{\mu }$ is the estimated cohort genotype mean vector computed as $\Sigma_{i=1}^{N}{\hat{w}}_{i}{\hat{\mu }}_{i}$. Second, normalize C to obtain the correlation matrix, Σ, by dividing each covariance by the product of the corresponding SNP genotype standard deviations. Third, to avoid false positive when cohort and panel ethnicities are not well matched (and, thus, weights might not sum to one), add a ridge adjustment by multiplying the diagonal elements of Σ with $\max(\Sigma_{i=1}^{N}{\hat{w}}_{i},\;1/\Sigma_{i=1}^{N}{\hat{w}}_{i})$.

To avoid ill-conditioned mixture correlation matrix due to the highly correlated LD structure, we add a second ridge adjustment, heuristically set to $\lambda =2/\sqrt{n}$ (where n is the sample size of the reference population), to the diagonal elements of Σ (Lee *et al.*, 2015; Pasaniuc *et al.*, 2014; Pickrell, 2014). Σ is subsequently used to impute two-tailed Z-scores of unmeasured SNPs using the conditional expectation formula for multivariate normal variates (Lee *et al.*, 2013). To obtain *Z*-scores, DISTMIX uses the square root of the imputation information to normalize (to a variance of one) the conditional expectations (Lee *et al.*, 2015; Pasaniuc *et al.*, 2014).

### 2.2 Assessment of the type I error rate of DISTMIX

To assess the Type I error rate and the accuracy of ethnicity weight estimation for the proposed method, we simulated, under the null hypothesis of no association (H_0_), five sets of 100 summary data sets of Ilumina 1 M autosomal SNPs from five different ethnicity combinations in 1KG: (i) 40% ASW + 60% GBR (Cohort 1), (ii) 60% CHB + 40% MXL (Cohort 2), (iii) 20% ASW + 30% CHB + 30% GBR + 20% MXL (Cohort 3), (iv) 30% CEU + 25% CHS + 5% PUR + 40% YRI (Cohort 4) and (v) 10% ASW + 15% CEU + 15% CHB + 12.5% CHS + 15% GBR + 10% MXL + 2.5% PUR + 20% YRI (Cohort 5) (See Supplementary Table S1 for abbreviations for ethnicities in 1KG and Section 1 in Supplementary Data for null summary data set simulations).

Using DISTMIX at its default settings (Supplementary Table S2) and 1KG (phase 1 release version 3 with 1092 subjects and minor AF (MAF) ≥ 0.5%), we imputed each null GWAS summary data from Cohorts 1-4. For summary data from Cohort 5, we only estimated ethnicity proportions. To assess the robustness of DISTMIX when the reference panel does not incorporate best-matching ethnicities, we also imputed simulated data sets from Cohort 3 using a subset of 1KG reference panel which excluded the relevant ethnicities (GBR and MXL). Based the DISTMIX H_0_ results, we estimated empirical Type I error rates across different nominal levels.

### 2.3 Comparison with genotype imputation method

We compared the performance of DISTMIX and the commonly used IMPUTE2/SHAPEIT (Delaneau *et al.*, 2012; Howie *et al.*, 2009) using all the 9 million autosomal SNPs summary statistics reported for PGC SCZ2 discovery phase (Ripke *et al.*, 2014), i.e. IMPUTE2 imputation information score ≥ 0.6 and MAF ≥ 1%. (We note that the filtering cutoff (0.6) on IMPUTE2 imputation information used in PGC SCZ2 discovery phase is slightly higher than the ones used in other studies.) From the combined discovery-replication analysis, PGC SCZ2 identified 105 autosomal LD independent association regions, defined as the regions containing all SNPs in LD ($r^{2}>0.6$) with the PGC SCZ2 top SNPs. For a conservative comparison, from these 9 million SNPs, we first deemed as ‘measured’ 675 K autosomal SNPs (hereafter referred to as PGC SCZ2 1 M) consisting only of Ilumina 1 M SNPs with information scores ≥ 0.95. On the basis of summary statistics for these 675 K SNPs, we re-imputed the remaining SNPs using DISTMIX at its default settings with the 1KG reference panel. To demonstrate the advantage of DISTMIX's weighting approach over simple selection of reference populations, we re-imputed the PGC SCZ2 1 M SNPs using DIST at the same default settings with two continental reference (European (EUR) and Asian (ASN)) populations from 1KG. Subsequently, both DISTMIX and DIST results were compared to PGC SCZ2 data. For both sets of results, we do not apply any post-imputation filtering based on imputation quality.

## 3 Results

### 3.1 Simulated data under the null hypothesis

Under the null hypothesis of no association, DISTMIX delivers accurate and reliable estimates for the weights of ethnicities from 1KG reference panel (Table 1). Mean values of the 100 estimated weight sets for all 5 simulated cohorts are very close to actual values. Even more, the accuracy of the weight estimates is remarkable, the standard deviation (SD) for any of these estimates falling below 0.2%. The increased complexity of cohort (Cohorts 3–5 in Table 1) does not diminish the estimation accuracy. When no perfectly matched ethnicities exist in the reference population, the weight of next closest ethnicities is increased (Cohort 3* in Table 1).

**Table 1.** Estimated weights (%) for 1KG ethnicities (see Supplementary Table S1 for abbreviations of ethnicities)

Cohort	Estimated weights (%)													
	ASW	CEU	CHB	CHS	CLM	FIN	GBR	IBS	JPT	LWK	MXL	PUR	TSI	YRI
*Mean mixing proportions of null data sets (for all estimates SD < 0.2%)*														
Cohort 1 40% ASW + 60% GBR	40	0	0	0	0	0	60	0	0	0	0	0	0	0
Cohort 2 60% CHB + 40% MXL	0	0	60	0	0	0	0	0	0	0	39.9	0	0	0
Cohort 3 20% ASW + 30% CHB + 30% GBR + 20% MXL	20	0	29.9	0	0	0	29.9	0	0	0	20	0	0	0
Cohort 3* 20% ASW + 30% CHB + 30% GBR + 20% MXL	22.3	12.1	30.3	0	16.3	6.4	-	0.5	1.3	0	-	7.8	2.9	0
Cohort 4 30% CEU + 25% CHS + 5% PUR + 40% YRI	0	30	0	25	0	0	0	0	0	0	0	5	0	40
Cohort 5 10% ASW + 15% CEU + 15% CHB + 12.5% CHS + 15% GBR + 10% MXL + 2.5% PUR + 20% YRI	10	15	15	12.5	0	0	15	0	0	0	10	2.5	0	20
*Mixing proportions of PGC SCZ2 data*														
PGC SCZ2	2	25.2	2.1	4	2.3	17.9	24.3	2.4	0.5	0	0	3.9	15.4	0

All cohorts use 1KG as the reference panel, except Cohort 3* which used 1KG without GBR and MXL.

**Fig. 1.**
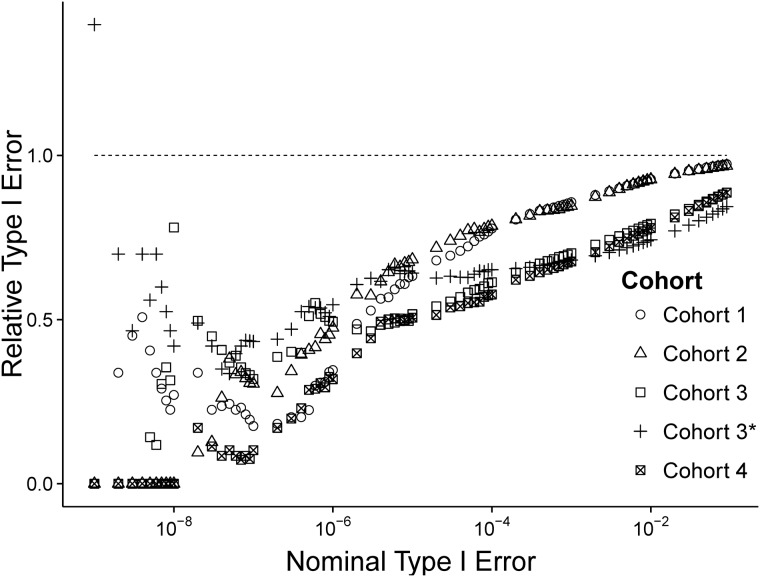
DISTMIX relative Type I error rate (the empirical Type I error rate divided by the nominal Type I error rate) as a function of the nominal Type I error rate and the null summary data used. Cohort 1, 40% ASW + 60% GBR; Cohort 2, 60% CHB + 40% MXL; Cohort 3, 20% ASW + 30% CHB + 30% GBR + 20% MXL; Cohort 4, 30% CEU + 25% CHS + 5% PUR + 40% YRI. All cohorts use 1KG as the reference panel, except Cohort 3* which used 1KG without GBR and MXL. The dashed line (at 1) denotes the nominal threshold for the relative Type I error rate

DISTMIX controls the Type I error rate at or below the nominal level for all simulated cohorts (Fig. 1). While some increase in the Type I error rates was observed when excluding two best-matching ethnicities from the reference population (Cohort 3*) (compared to Cohort 3 which used the reference panel containing all best-matching ethnicities), DISTMIX still maintains the Type I errors at or below the nominal level (Fig. 1).

### 3.2 Comparison with IMPUTE2 using PGC SCZ2 data

The estimated weights of 1KG ethnicities for PGC SCZ 1 M are also realistic (last row of Table 1). The estimated proportions for European (25.2% for CEU; 17.9% for FIN; 24.3% for GBR; 2.4% for IBS; 15.4% for TSI) and Asian cohorts (0.5% for JPT; 2.1% for CHB; 4% for CHS) are close to the actual proportion of European (∼91%) and Asian cohort subjects (∼%1 for Japan; ∼5% for Chinese ancestry (Singapore and China) in PGC SCZ2. Small non-zero weights for ASW, CLM and PUR might capture some of the European background from these heavily admixed American populations.

**Fig. 2.**
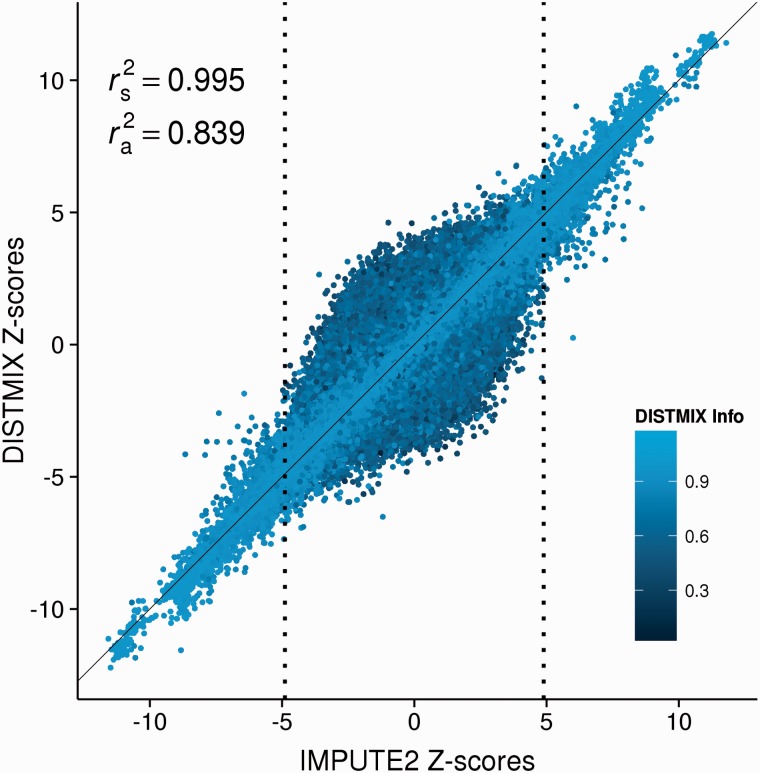
DISTMIX Z-scores as a function of IMPUTE2 Z-scores from PGC SCZ2 discovery phase and DISTMIX imputation information. The vertical dotted lines represent the suggestive thresholds for PGC SCZ2 discovery phase (IMPUTE2 *P*-value < 1 × 10^−6^). *r_s_^2^*, the squared correlation coefficient (*r^2^*) between DISTMIX and IMPUTE2 Z-scores for the suggestive PGC SCZ2 SNPs; *r_a_^2^*, *r^2^* between two predictions for all SNPs

The imputed DISTMIX and IMPUTE2 statistics for PGC SCZ2 discovery phase behave quite similarly (Fig. 2 and Supplementary Fig. S1). The analysis of 7 425 593 markers imputed by DISTMIX shows that DISTMIX prediction is fairly comparable to IMPUTE2 (Fig. 2), the squared correlation coefficient ($r^{2}$) between the two predictions being 83.9%. More importantly, for the reported 17 029 suggestive markers (IMPUTE2 *P*-value<1 × 10^−^^6^), $r^{2}$ between the two predictions increases to 99.5%. (When compared to DISTMIX, DIST using EUR + ASN as a reference population performed poorly (Supplementary Fig. S2): $r^{2}$ between DIST and IMPUTE2 Z-scores was 79.4% for all SNPs and 98.1% for suggestive signals. This result shows that for cosmopolitan cohorts like PGC SCZ2, DISTMIX's weighting approach outperforms approaches of simple unweighted selection of reference populations.) Out of 995/1631 SNPs having significant (*P*-value < 5 × 10^−^^8^)/suggestive (*P*-value < 1 × 10^−^^6^) signals for IMPUTE2 but not for DISTMIX (Fig. 2), 975/1174 (98%/72%) SNPs are in or near (±250 Kb) the 105 LD independent autosomal association regions reported by PGC SCZ2. Among them, 466/606 SNPs are from the extended MHC association region (26–34 Mb). On the other hand, out of 1045/1916 SNPs with significant/suggestive association signals for DISTMIX but not for IMPUTE2, 1010/1564 (96.7%/81.6%) SNPs are from in or near the association locus and 398/445 SNPs are from the MHC region.

**Fig. 3.**
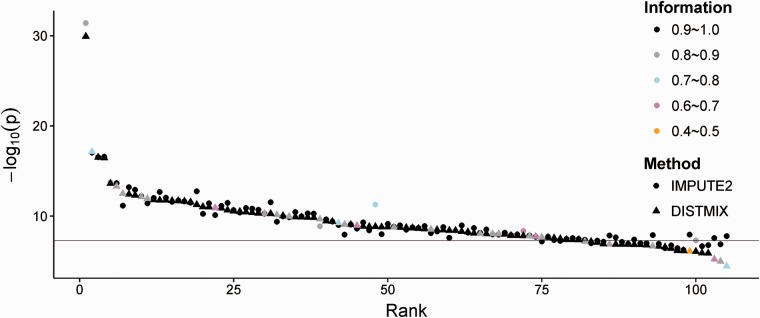
−log_10_(*p*) for the 105 LD independent autosomal association PGC SCZ2 SNPs as a function of the rank of significance for DISTMIX *P*-values, imputation information and imputation method used

For a more detailed performance assessment, we also compared the top p-values of DISTMIX and IMPUTE2 for the 105 significant autosomal association regions reported by PGC SCZ2 (Fig. 3 and Supplementary Table S3). While IMPUTE2 applied to PGC SCZ2 discovery phase identified 88 (83.8%) statistically significant regions out of these 105 regions, DISTMIX successfully detected 81 (77.1%) as significant, including two regions not identified by IMPUTE2. However, the regions not detected by just one of the two methods yielded, for the smaller signal, p-values just below the significance threshold (Fig. 3). When compared to IMPUTE2, most of the apparent conservativeness of DISTMIX is likely due to the LD estimation using the ridge penalty ($\lambda =2/\sqrt{n}$) for the small 1KG reference panel used for imputation. We expect this difference to become negligible when using the very large next generation reference panels. (Note that the difference in signal detection is not technically a power loss for DISTMIX, since we compare the results of each imputation method with PGC2 IMPUTE2 signals and not with the true signals.)

On a Linux cluster with 24 computation nodes, each having 4 x Intel Xeon 6 core 2.67 Ghz processor and 64 GB of RAM, the imputation of PGC SCZ2 1 M was performed in parallel using 40 cores (one core per autosome chromosome arm). The running time and peak memory usage were slightly under 12 hours and 1 GB, respectively. Remarkably, the running time translates to a single core computation time of less than one week.

## 4 Conclusions

DIST and ImpG assume that the genotypes and association statistics have identical correlation structures. However, while the assumption is reasonable for homogeneous cohorts, it might not be met when there are relevant covariates which confound genotypes, e.g. ancestry principal components in ethnically mixed studies. To adequately analyze such cohorts, we propose DISTMIX, a very fast and novel method/software for directly imputing summary statistics of untyped makers from cosmopolitan cohorts without using subject-level genotype data. The proposed method (i) uses mixture proportions for each ethnicity in a reference panel (e.g. 1KG) either (a) provided by the user or (b) estimated based on the in-cohort estimated AFs of common variants, (ii) uses these proportions to determine the cohort LD as a mixture of the LDs of the ethnicities from the reference panel and (iii) uses the mixture LD in DIST procedure to impute statistics at untyped variants.

As shown by our simulation and empirical studies, for cosmopolitan cohorts, DISTMIX (i) accurately estimates in-study weights of ethnicities from a reference panel (when they are not provided by the user), (ii) maintains the Type I error rate at or below the nominal level, (iii) delivers comparable imputation accuracy to commonly used genotype imputation methods while (iv) dramatically reducing computational needs. Moreover, given that the relatedness between subjects in a cohort does not affect the estimated mixture LD, DISTMIX can be used ‘as-is’ for meta-analyses containing family data.

Compared to summary statistics based imputation methods like DISTMIX, genotype imputation methods offer more flexibility to researchers; once haplotype phasing and genotype imputation are done for a sample cohort, researchers can conduct different GWASs using different phenotypes and covariates without re-imputation. While the current DISTMIX version imputes only one set of summary values, given that the most computer intensive part is the estimation of the correlation matrix, we believe that a future version simultaneously imputing Z-score for multiple traits is attainable with minimal effort.

Due to the emergence of very large reference populations such as the Haplotype Reference Consortium (HRC) including more than 30 000 subjects at over 50 million SNPs (Kretzschmar *et al.*, 2014) (http://www.haplotype-reference-consortium.org), imputation procedures will be required to both impute new studies and re-impute previously published GWASs. This process will require a sudden increase in processing capabilities due to these larger panel sizes. For example, even after an impressive ∼20× speed improvement, the genotype imputation using the HRC panel, will still be around 2 times slower than the same imputation using present methods with the 1KG panel (Fuchsberger *et al.*, 2014). At these reference panel sizes, DISTMIX will also have much longer running times. Given that the most computer intensive part of DISTMIX imputation is the reference panel-based computation of the correlation matrix, in future DISTMIX versions we plan to pre-compute, and store in a database, the local correlation matrix of genetic regions by each ethnic group (Pasaniuc *et al.*, 2014). Thus, DISTMIX using the pre-computed LD matrices will dramatically reduce the running time associated with future large reference panels. Moreover, such an approach has the added advantage of making the computational burden of summary statistic imputation practically invariant to the sample size of the reference panel. This invariability will be a useful feature with the likely increase in study sizes.

When compared to genotype imputation methods e.g. IMPUTE2 and MACH (Li *et al.*, 2010), due to its sample size dependent ridge estimate ($\lambda =2/\sqrt{n}$), DISTMIX might deliver somewhat conservative results for the rather small existing panels, such as 1KG used in our paper (Fig. 3). (Its conservativeness is likely to be more pronounced at lower imputation information). However, the ridge estimate, and thus conservativeness, will be greatly diminished soon, as the size of reference panels is increased by almost two orders of magnitude (see the HRC panel above). To reduce the computational runtime and the complexity of our implementation, DISTMIX is written in C++ with open-source libraries and publicly available online.

## Funding

This work was supported by R25DA026119 (D.L.), MH100560 (S.A.B. and B.P.R.), AA022717 (S.A.B., V.I.V. and V.S.W.) and 1P50AA022537 (S.A.B. and B.P.R.).

*Conflict of Interest*: none declared.

## Supplementary Material

Supplementary Data
