# Supplementary material for: DISTMIX: direct imputation of summary statistics for unmeasured SNPs from mixed ethnicity cohorts
Source: Bioinformatics. 2015 Jun 9;31(19):3099–104. doi: 10.1093/bioinformatics/btv348 (PMC4576696; doi:10.1093/bioinformatics/btv348)
Supplement: Supplementary Data [file supp_31_19_3099__index.html]

DISTMIX: Direct imputation of summary statistics for unmeasured SNPs from mixed ethnicity cohorts — DISTMIX: direct imputation of summary statistics for unmeasured SNPs from mixed ethnicity cohorts — DISTMIX: direct imputation of summary statistics for unmeasured SNPs from mixed ethnicity cohorts — Supplementary Data 

# DISTMIX: direct imputation of summary statistics for unmeasured SNPs from mixed ethnicity cohorts

## Supplementary Data

files

- Supplementary Data - pdf file
