# Supplementary material for: DISTMIX: direct imputation of summary statistics for unmeasured SNPs from mixed ethnicity cohorts
Source: Bioinformatics. 2015 Jun 9;31(19):3099–104. doi: 10.1093/bioinformatics/btv348 (PMC4576696; doi:10.1093/bioinformatics/btv348)
Supplement: Supplementary Data [file supp_btv348_distmix_supplementary_data.pdf]

Supplementary Data

Table S1. 1000 Genome population abbreviations. AFR is an abbreviation for African; AMR for Admixed American; ASN for East Asian; EUR for European.

Population Abbreviation	Number of Subjects	Super Population	Population Description
ASW	61	AFR	African Ancestry in Southwest US
CEU	85	EUR	Utah residents (CEPH) with Northern and Western European ancestry
CHB	97	ASN	Han Chinese in Beijing, China
CHS	100	ASN	Southern Han Chinese
CLM	60	AMR	Colombian in Medellin, Colombia
FIN	93	EUR	Finnish in Finland
GBR	89	EUR	British in England and Scotland
IBS	14	EUR	Iberian populations in Spain
JPT	89	ASN	Japanese in Tokyo, Japan
LWK	97	AFR	Luhya in Webuye, Kenya
MXL	66	AMR	Mexican Ancestry from Los Angeles, USA
PUR	55	AMR	Puerto Rican in Puerto Rico
TSI	98	EUR	Toscani in Italia
YRI	88	AFR	Yoruba in Ibadan, Nigeria

1. Simulating Ilumina 1M GWAS summary statistics under the null hypothesis (H_0) of no association

We simulated (under H_0) 100 realistic GWAS summary data sets of Illumina 1M (http://www.illumina.com) autosomal SNPs for five combinations of ethnicities in the 1000 Genomes population: 1) 40% ASW + 60% GBR (Cohort 1), 2) 60% CHB + 40% MXL (Cohort 2), 3) 20% ASW + 30% CHB + 30% GBR + 20% MXL (Cohort 3), 4) 30% CEU + 25% CHS + 5% PUR + 40% YRI (Cohort 4) and 5) 10% ASW + 15% CEU + 15% CHB + 12.5% CHS + 15% GBR + 10% MXL + 2.5% PUR + 20% YRI (Cohort 5). For each of these data sets, we simulated the genotype data of 10,000 subjects by i) randomly drawing with replacement a subject's genotypes from each combination of ethnicities and ii) retaining as GWAS (measured) autosomal SNPs only those found on Illumina 1M chip. The phenotype of each subject was simulated as a random standard normal variable. The summary statistics were obtained by testing for association between the simulated SNP genotypes and phenotypes using linear regression.

Table S2. Command line options currently available in DISTMIX software.

Option	Short Flag	Parameter	Default	Description
--version	-v	none	none	Prints version information
--help	-h	none	none	Outputs a full description of all DISTMIX options
--reference	-r	filename	none	The filename of the reference panel data
--referenceIndex	-i	filename	none	The filename of the reference panel index data
--output	-o	filename	out.distmix	The output filename. This file has a header and is space-delimited. Rows correspond to SNPs and columns to variables
--chromosome	-c	positive integer between 1 and 22 or string such as "1p" or "2q"	none	The chromosome number (or the chromosome number and arm, e.g., 1q, 22p)
--windowSize	-n	decimal	1.0	The size of the DISTMIX prediction window* (Mb)
--wingSize	-m	decimal	0.5	The size of the area (wing) flanking the left and right of the DISTMIX prediction window (Mb)
--populationWeight	-w	filename	none	The filename of the population weight data

* DISTMIX imputes unmeasured SNP statistics within the prediction window based on measured SNP statistics within the extended window (i.e. the prediction window with flanking regions (wings) on both sides.

Figure S1. Manhattan plots of the $-\log_{10}(p)$ for PGC SCZ2 discovery phase imputed by IMPUTE2/SHAPEIT (top) and DISTMIX (bottom). The horizontal line represents the genome wide significance threshold ($p = 5 \times 10^{-8}$).

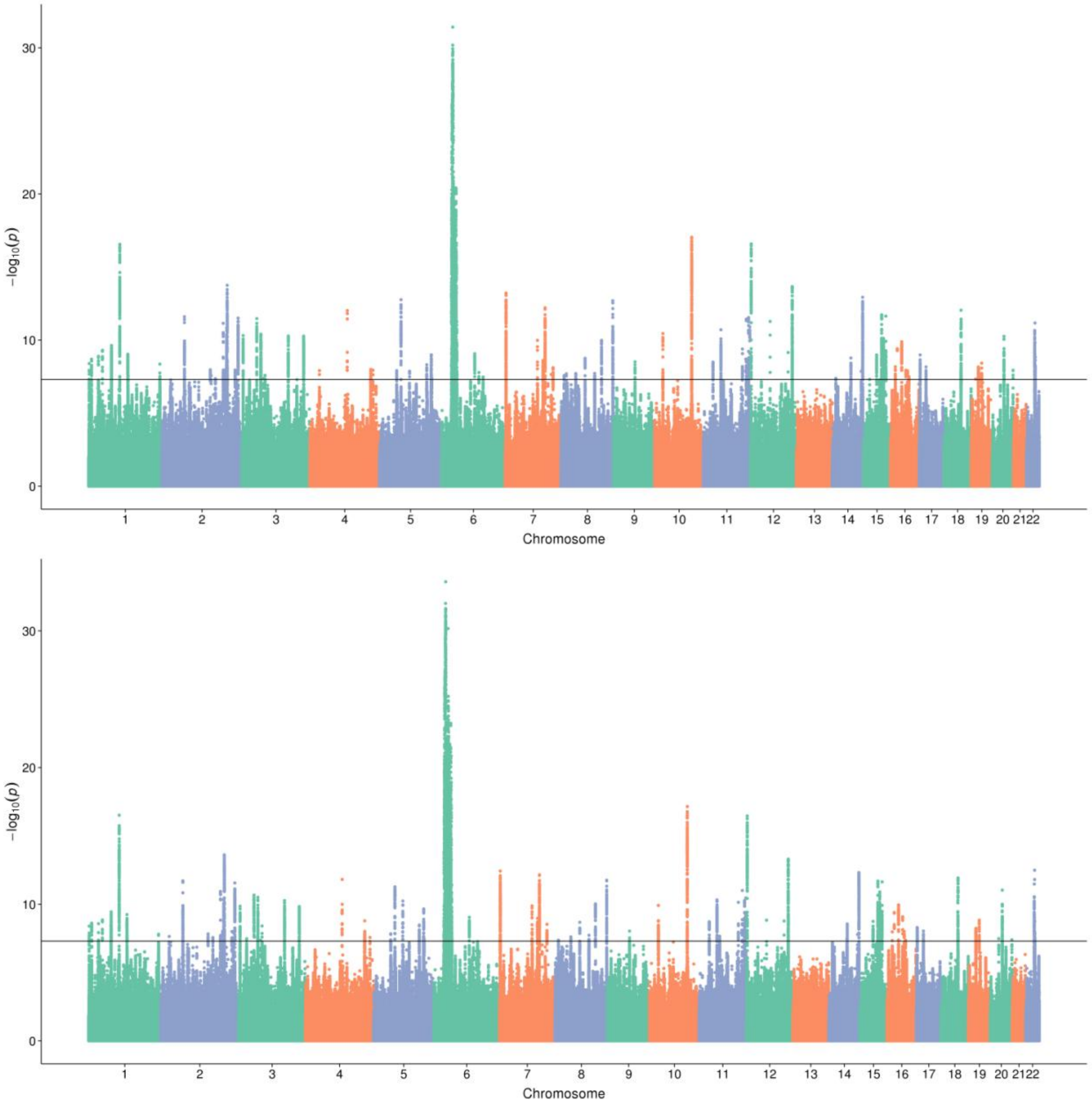

Figure S2. DIST Z-scores as a function of IMPUTE2 Z-scores from PGC2 SCZ discovery phase and DIST imputation information. EUR+ASN from 1KG was used as a reference panel. The vertical dotted lines represent the suggestive thresholds for PGC SCZ2 discovery phase (IMPUTE2 p-value < 1×10^{-6}). r_s^2, the squared correlation coefficient (r^2) between DIST and IMPUTE2 Z-scores for the suggestive PGC SCZ2 SNPs; r_a^2, r^2 between two predictions for all SNPs.

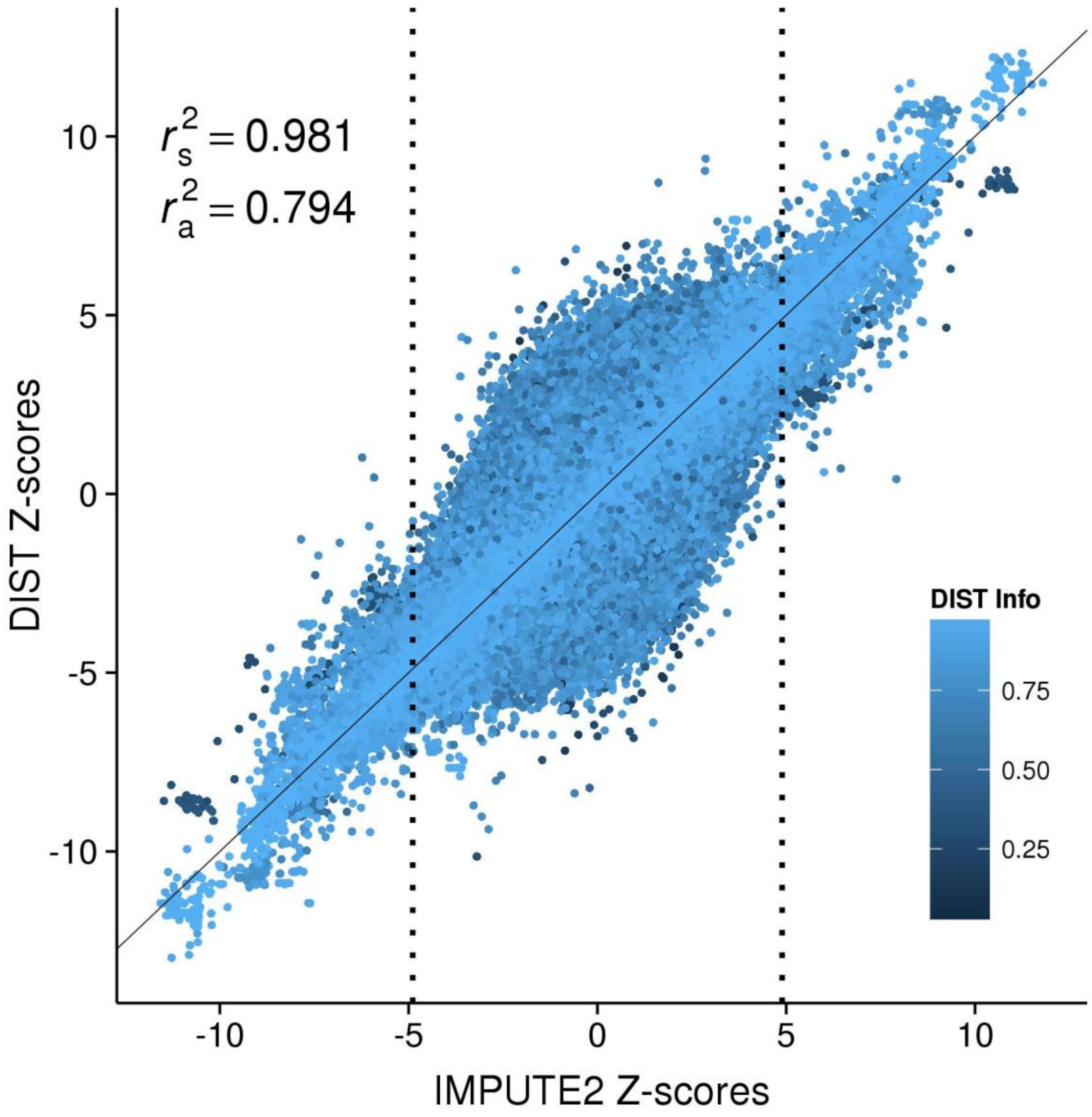

Table S3. Comparison of summary statistics of top discovery SNPs at the 105 significant autosomal association regions imputed by IMPUTE2 (obtained from PGC SCZ2 discovery phase) and imputed by DISTMIX using PGC SCZ2 1M data. In **bold** SNPs imputed by DISTMIX. Chr, chromosome number; Position, base pair position; A12, the SNP alleles having the first allele as the reference allele; P, p-value; Info, imputation information score; A1, the reference allele; A2, the alternative allele.

IMPUTE2 (PGC SCZ2)							DISTMIX								
Rank	Chr	Position	SNP ID	A12	P	Info	Rank	Chr	Position	SNP ID	A1	A2	P	Info
59	1	2,387,101	rs4648845	TC	4.03E-09	0.827	61	1	2,387,101	rs4648845	T	C	4.03E-09	1
54	1	8,424,984	chr1_8424984_D	I2D	2.03E-09	0.989	**55**	**1**	**8,448,812**	**rs3050831**	**A**	**AAT**	**2.44E-09**	**0.937**
48	1	30,433,951	rs1498232	TC	1.28E-09	0.985	56	1	30,437,268	rs6694545	G	A	2.64E-09	1
42	1	44,100,084	rs11210892	AG	4.97E-10	0.997	**46**	**1**	**44,097,530**	**rs2970610**	**C**	**T**	**1.42E-09**	**0.974**
39	1	73,768,366	rs12129573	AC	2.35E-10	0.996	40	1	73,816,564	rs1160682	T	C	3.62E-10	1
4	1	98,501,984	rs1702294	TC	2.79E-17	1	3	1	98,502,340	rs1782810	A	G	3.07E-17	1
45	1	150,031,490	rs140505938	TC	9.34E-10	0.991	**42**	**1**	**150,110,056**	**rs56397877**	**A**	**G**	**5.77E-10**	**0.733**
97	1	177,280,121	rs6670165	TC	1.16E-07	0.994	94	1	177,271,313	rs16851037	A	G	2.28E-07	1
100	1	207,977,083	rs7523273	AG	1.61E-07	0.992	102	1	207,957,555	rs7541230	C	T	1.31E-06	1
60	1	243,555,105	rs77149735	AG	4.40E-09	0.607	**72**	**1**	**243,645,203**	**rs12751210**	**G**	**C**	**1.58E-08**	**0.912**
16	2	57,987,593	rs11682175	TC	2.54E-12	0.991	**15**	**2**	**57,961,602**	**rs13011472**	**G**	**C**	**1.95E-12**	**0.953**
99	2	72,361,505	rs3768644	AG	1.30E-07	0.966	**104**	**2**	**72,361,505**	**rs3768644**	**A**	**G**	**1.06E-05**	**0.866**
67	2	146,436,222	chr2_146436222_I	I2D	1.07E-08	0.978	71	2	146,425,531	rs16825349	G	A	1.49E-08	1
81	2	149,429,178	chr2_149429178_D	I2D	2.62E-08	0.94	**103**	**2**	**149,462,958**	**rs145171722**	**A**	**G**	**6.24E-06**	**0.648**
87	2	162,845,855	rs2909457	AG	4.38E-08	1	**77**	**2**	**162,845,855**	**rs2909457**	**A**	**G**	**2.73E-08**	**0.946**
24	2	185,601,420	rs11693094	TC	7.13E-12	0.977	**23**	**2**	**185,778,428**	**rs1344706**	**C**	**A**	**1.29E-11**	**0.937**
68	2	193,848,340	rs59979824	AC	1.08E-08	0.929	**67**	**2**	**194,028,079**	**rs1882273**	**C**	**G**	**9.37E-09**	**0.856**
26	2	198,304,577	rs6434928	AG	1.48E-11	0.955	**28**	**2**	**198,474,908**	**rs77368480**	**T**	**C**	**4.17E-11**	**0.91**
84	2	200,164,252	rs6704641	AG	3.40E-08	0.992	**79**	**2**	**200,221,052**	**rs66560483**	**TA**	**T**	**4.20E-08**	**0.917**
5	2	200,825,237	chr2_200825237_I	I2D	1.78E-14	0.877	**5**	**2**	**200,715,388**	**rs2949006**	**G**	**T**	**2.45E-14**	**0.943**
69	2	225,391,296	rs11685299	AC	1.11E-08	0.988	**68**	**2**	**225,395,641**	**rs200533090**	**A**	**AAAC**	**9.88E-09**	**0.887**
18	2	233,592,501	rs6704768	AG	3.15E-12	0.995	**18**	**2**	**233,716,421**	**rs4973563**	**T**	**C**	**2.78E-12**	**0.952**
31	3	2,547,786	rs17194490	TG	4.87E-11	0.973	37	3	2,537,916	rs17194476	T	C	1.40E-10	1
90	3	17,859,366	rs4330281	TC	5.51E-08	0.992	**78**	**3**	**17,859,855**	**rs4908985**	**C**	**T**	**3.30E-08**	**0.963**
19	3	36,858,583	rs75968099	TC	3.39E-12	0.98	24	3	36,862,980	rs4624519	T	C	2.15E-11	1
30	3	52,845,105	rs2535627	TC	3.96E-11	0.986	**26**	**3**	**52,845,105**	**rs2535627**	**C**	**T**	**3.10E-11**	**0.95**
80	3	63,833,050	rs832187	TC	2.58E-08	0.985	**60**	**3**	**63,833,387**	**rs34196118**	**T**	**TG**	**3.93E-09**	**0.908**
32	3	136,288,405	rs7432375	AG	5.27E-11	0.988	**30**	**3**	**136,247,337**	**.**	**CCG**	**C**	**5.45E-11**	**0.88**
33	3	180,594,593	chr3_180594593_I	I2D	5.35E-11	0.921	38	3	180,615,583	rs34133935	C	T	1.48E-10	1

72	4	23,423,603	rs215411	AT	1.22E-08	0.993	**93**	**4**	**23,377,121**	**rs215483**	**A**	**G**	**2.16E-07**	**0.874**
13	4	103,146,890	rs35518360	AT	9.56E-13	0.922	12	4	103,188,709	rs13107325	T	C	1.54E-12	1
66	4	170,626,552	rs10520163	TC	1.02E-08	0.995	**49**	**4**	**170,550,327**	**rs138997528**	**T**	**TCTGTATCTTGA**	**1.64E-09**	**0.942**
70	4	176,861,301	rs1106568	AG	1.15E-08	1	**99**	**4**	**176,851,585**	**.**	**G**	**GT**	**7.89E-07**	**0.481**
73	5	45,364,875	rs1501357	TC	1.24E-08	0.98	70	5	45,301,035	rs9292918	G	T	1.47E-08	1
9	5	60,598,543	rs4391122	AG	1.73E-13	0.983	19	5	60,586,625	rs4352559	C	T	5.39E-12	1
74	5	88,746,331	rs16867576	AG	1.36E-08	0.944	**86**	**5**	**88,744,550**	**rs7701188**	**A**	**G**	**1.34E-07**	**0.831**
95	5	109,036,066	rs4388249	TC	1.03E-07	0.992	**83**	**5**	**109,190,934**	**rs200733039**	**T**	**TTC**	**6.75E-08**	**0.911**
61	5	137,851,192	rs3849046	TC	4.83E-09	0.993	**58**	**5**	**137,841,064**	**rs35191349**	**G**	**GCTC**	**3.32E-09**	**0.955**
104	5	140,143,664	chr5_140143664_I	I12D	3.60E-07	1	**97**	**5**	**140,136,468**	**rs2563269**	**T**	**G**	**6.95E-07**	**0.96**
49	5	152,177,121	rs111294930	AG	1.31E-09	0.821	**39**	**5**	**152,589,220**	**rs13168705**	**G**	**A**	**2.22E-10**	**0.828**
85	5	153,680,747	rs11740474	AT	3.94E-08	0.97	**90**	**5**	**153,687,040**	**rs35495653**	**T**	**C**	**1.43E-07**	**0.96**
1	6	28,712,247	rs115329265	AG	3.86E-32	0.833	1	6	28,689,672	rs116594362	A	C	1.17E-30	1
92	6	73,155,701	rs1339227	TC	6.86E-08	0.982	**85**	**6**	**73,161,604**	**rs11968261**	**T**	**C**	**1.25E-07**	**0.935**
44	6	84,280,274	chr6_84280274_D	I2D	8.57E-10	0.924	**44**	**6**	**84,373,058**	**rs60730**	**A**	**G**	**8.99E-10**	**0.966**
76	6	96,459,651	rs117074560	TC	1.66E-08	0.912	**105**	**6**	**96,459,651**	**rs117074560**	**T**	**C**	**3.52E-05**	**0.714**
7	7	2,025,096	chr7_2025096_I	DI3	6.12E-14	0.998	8	7	2,017,445	rs6461049	T	C	3.73E-13	1
103	7	24,747,494	chr7_24747494_D	DI3	3.59E-07	0.974	**95**	**7**	**24,717,969**	**rs79210963**	**C**	**T**	**2.76E-07**	**0.946**
36	7	86,427,626	rs12704290	AG	1.04E-10	0.992	**36**	**7**	**86,427,626**	**rs12704290**	**A**	**G**	**1.32E-10**	**0.939**
55	7	104,929,064	rs6466055	AC	2.46E-09	0.997	**45**	**7**	**104,960,307**	**rs6466058**	**T**	**C**	**1.10E-09**	**0.608**
105	7	110,048,893	rs211829	TC	5.47E-07	0.998	98	7	110,074,276	rs9656169	T	C	7.04E-07	1
11	7	110,898,915	rs13240464	TC	6.16E-13	0.994	**10**	**7**	**110,921,315**	**rs71544722**	**G**	**A**	**7.06E-13**	**0.808**
79	7	131,567,263	rs7801375	AG	2.26E-08	0.991	**87**	**7**	**131,567,263**	**rs7801375**	**G**	**A**	**1.36E-07**	**0.95**
65	7	137,074,844	rs3735025	TC	7.75E-09	0.997	**57**	**7**	**137,039,670**	**rs34093284**	**GA**	**G**	**2.93E-09**	**0.842**
82	8	4,180,844	rs10503253	AC	2.69E-08	1	**80**	**8**	**4,184,179**	**rs10046759**	**G**	**C**	**4.28E-08**	**0.956**
78	8	27,442,127	rs73229090	AC	1.95E-08	0.942	**76**	**8**	**27,424,696**	**rs59724122**	**T**	**C**	**2.46E-08**	**0.942**
52	8	60,700,469	rs6984242	AG	1.76E-09	1	54	8	60,694,647	rs867743	A	G	2.11E-09	1
77	8	89,588,626	rs7819570	TG	1.90E-08	0.998	**74**	**8**	**89,592,168**	**rs36012446**	**C**	**T**	**1.88E-08**	**0.669**
37	8	111,485,761	rs36068923	AG	1.05E-10	0.996	**33**	**8**	**111,600,728**	**rs6990323**	**T**	**G**	**9.46E-11**	**0.919**
10	8	143,312,933	rs4129585	AC	2.03E-13	0.991	13	8	143,309,504	rs10098073	A	C	1.74E-12	1
56	9	84,739,941	rs11139497	AT	3.09E-09	0.969	66	9	84,788,136	rs2788116	A	C	9.33E-09	1
29	10	18,745,105	rs7893279	TG	3.56E-11	1	35	10	18,733,293	rs4748466	G	A	1.23E-10	1
2	10	104,612,335	rs11191419	AT	9.24E-18	0.951	**2**	**10**	**104,957,628**	**rs200399577**	**A**	**AT**	**7.18E-18**	**0.716**
57	11	24,403,620	rs11027857	AG	3.21E-09	0.996	**53**	**11**	**24,404,054**	**rs10834332**	**G**	**C**	**1.92E-09**	**0.969**
27	11	46,350,213	chr11_46350213_D	I2D	1.97E-11	0.936	**29**	**11**	**46,350,213**	**rs61126341**	**A**	**AG**	**4.81E-11**	**0.914**
91	11	57,510,294	rs9420	AG	6.65E-08	1	**75**	**11**	**57,464,944**	**rs200927786**	**A**	**AT**	**2.22E-08**	**0.884**
101	11	109,378,071	rs12421382	TC	1.72E-07	0.99	**96**	**11**	**109,389,471**	**rs1808843**	**C**	**T**	**3.87E-07**	**0.955**
41	11	113,392,994	rs2514218	TC	4.09E-10	0.99	**32**	**11**	**113,407,114**	**rs3133388**	**A**	**G**	**7.28E-11**	**0.848**
53	11	123,394,636	rs77502336	CG	2.01E-09	0.968	**64**	**11**	**123,394,636**	**rs77502336**	**C**	**G**	**7.94E-09**	**0.938**
21	11	124,613,957	rs55661361	AG	3.68E-12	0.985	**21**	**11**	**124,612,932**	**rs12293670**	**G**	**A**	**9.85E-12**	**0.944**
17	11	130,718,630	rs10791097	TG	2.88E-12	0.995	**31**	**11**	**130,717,256**	**rs4366492**	**C**	**T**	**5.45E-11**	**0.961**

25	11	133,822,569	rs75059851	AG	1.23E-11	0.973	27	11	133,827,733	rs3802924	C	A	3.50E-11	1
3	12	2,344,960	rs2007044	AG	2.63E-17	0.992	**4**	**12**	**2,344,960**	**rs2007044**	**G**	**A**	**3.49E-17**	**0.952**
93	12	29,917,265	rs679087	AC	7.06E-08	0.975	88	12	29,928,388	rs302321	A	C	1.38E-07	1
22	12	57,622,371	rs12826178	TG	5.30E-12	0.741	48	12	57,569,478	rs12814239	T	C	1.48E-09	1
96	12	92,246,786	rs4240748	CG	1.03E-07	0.979	91	12	92,248,851	rs7294593	G	A	1.46E-07	1
94	12	103,596,455	rs10860964	TC	9.92E-08	1	92	12	103,581,985	rs10860955	G	A	1.66E-07	1
43	12	110,723,245	rs4766428	TC	7.09E-10	0.941	**50**	**12**	**110,723,245**	**rs4766428**	**T**	**C**	**1.68E-09**	**0.917**
6	12	123,665,113	rs2851447	CG	2.19E-14	0.994	**6**	**12**	**123,682,081**	**rs1716180**	**A**	**G**	**5.04E-14**	**0.834**
86	14	30,190,316	rs2068012	TC	4.14E-08	0.973	**82**	**14**	**30,190,316**	**rs2068012**	**C**	**T**	**6.63E-08**	**0.853**
51	14	72,417,326	rs2332700	CG	1.69E-09	0.981	**59**	**14**	**72,418,321**	**rs12891745**	**T**	**C**	**3.61E-09**	**0.949**
75	14	99,719,219	rs2693698	AG	1.38E-08	0.959	**73**	**14**	**99,719,219**	**rs2693698**	**G**	**A**	**1.63E-08**	**0.899**
8	14	104,046,834	rs12887734	TG	1.17E-13	0.966	9	14	104,053,764	rs2403197	T	C	4.84E-13	1
88	15	40,567,237	rs56205728	AG	4.92E-08	0.887	**100**	**15**	**40,570,766**	**rs143550864**	**A**	**AAAAT**	**8.48E-07**	**0.91**
46	15	61,854,663	rs12903146	AG	1.04E-09	0.998	52	15	61,862,802	rs8042151	A	G	1.80E-09	1
89	15	70,589,272	rs12148337	TC	5.33E-08	0.999	84	15	70,576,138	rs1971791	A	G	8.52E-08	1
14	15	78,908,032	rs8042374	AG	1.87E-12	1	16	15	78,894,759	rs3743078	G	C	2.03E-12	1
35	15	84,706,461	rs950169	TC	7.62E-11	1	**22**	**15**	**85,052,366**	**rs141430008**	**C**	**T**	**1.27E-11**	**0.662**
15	15	91,426,560	rs4702	AG	2.30E-12	0.905	17	15	91,426,560	rs4702	A	G	2.30E-12	1
62	16	9,946,319	rs9922678	AG	6.72E-09	1	69	16	9,940,293	rs9926303	T	C	1.06E-08	1
40	16	13,749,859	rs7405404	TC	3.93E-10	0.997	41	16	13,753,384	rs8055219	A	G	4.01E-10	1
38	16	29,939,877	rs12691307	AG	1.30E-10	0.921	**34**	**16**	**29,935,505**	**rs11644809**	**A**	**G**	**1.11E-10**	**0.825**
71	16	58,681,393	rs12325245	AT	1.15E-08	0.953	**43**	**16**	**58,681,704**	**rs77101091**	**A**	**C**	**8.29E-10**	**0.888**
83	16	68,189,340	rs8044995	AG	3.27E-08	1	**81**	**16**	**68,285,847**	**rs1975802**	**G**	**A**	**4.30E-08**	**0.955**
47	17	2,208,899	rs4523957	TG	1.04E-09	0.956	**62**	**17**	**2,161,908**	**rs139504533**	**G**	**GCAA**	**5.11E-09**	**0.971**
63	17	17,958,402	rs8082590	AG	6.84E-09	0.954	**65**	**17**	**17,956,459**	**rs11658257**	**C**	**G**	**9.16E-09**	**0.88**
12	18	53,200,117	rs9636107	AG	9.09E-13	0.995	**14**	**18**	**53,200,117**	**rs9636107**	**G**	**A**	**1.77E-12**	**0.944**
20	18	53,533,189	rs72934570	TC	3.67E-12	0.981	**11**	**18**	**53,572,922**	**rs72936305**	**C**	**A**	**1.20E-12**	**0.859**
64	19	19,478,022	rs2905426	TG	6.92E-09	0.935	**63**	**19**	**19,657,632**	**rs7245983**	**C**	**A**	**5.86E-09**	**0.963**
58	19	30,987,423	rs2053079	AG	3.79E-09	0.987	**47**	**19**	**31,038,995**	**rs1469705**	**C**	**T**	**1.47E-09**	**0.914**
102	19	50,091,199	rs56873913	TG	2.19E-07	0.944	**101**	**19**	**50,091,464**	**rs10406941**	**C**	**A**	**1.27E-06**	**0.984**
34	20	37,453,194	rs6065094	AG	5.52E-11	1	**20**	**20**	**37,453,194**	**rs6065094**	**G**	**A**	**9.28E-12**	**0.963**
98	20	48,131,036	rs7267348	TC	1.18E-07	0.993	89	20	48,130,628	rs6019879	C	T	1.39E-07	1
28	22	39,987,017	chr22_39987017_D	I2D	2.20E-11	0.956	25	22	40,000,313	rs5995756	C	T	2.84E-11	1
23	22	41,587,556	rs9607782	AT	6.76E-12	0.944	**7**	**22**	**41,587,556**	**rs9607782**	**A**	**T**	**3.25E-13**	**0.893**
50	22	42,603,814	rs6002655	TC	1.48E-09	0.948	**51**	**22**	**42,571,028**	**rs760648**	**A**	**G**	**1.77E-09**	**0.836**
